# Traditional Chinese medicine paraffin therapy: an evidence-based overview from a modern medicine perspective

**DOI:** 10.1186/s13020-022-00662-z

**Published:** 2022-09-14

**Authors:** Wenxi Yan, Lanping Liu, Tao Yang, Xiaochen Yang

**Affiliations:** 1 Department of Acupuncture, China Academy of Chinese Medical Sciences, Guang’Anmen Hospital, Beijing, 100053 China; 2 Department of Cardiology and Health Care, China Academy of Chinese Medical Sciences, Guang’Anmen Hospital, Beijing, 100053 China

## Abstract

External therapy of traditional Chinese medicine and paraffin therapy are both traditional Chinese forms of treatment. In recent years, external use of traditional Chinese medicine combined with paraffin therapy, which involves combining meridians, acupoints, drugs, and hyperthermia, has demonstrated great effectiveness in treating certain conditions. An overview of traditional Chinese medicine paraffin therapy (TCMPT) is provided by this article. Additionally, this article describes a new classification of TCMPT, mechanism of action, clinical treatment, indications contraindications and adverse events reports.

## Introduction and brief history

In recent years, ‘traditional Chinese medicine non-oral drug therapy’ (TCMNDT) received widespread attention because of its wide range of adaptive treatment, targeted therapy that can quickly and effectively alleviate the patient' s pain and other advantages [1]. The proportion of TCMNDT in treatment has been included in the Healthy China 2021–2022 Assessment Program [2]. At the same time, the state will lead the development of ‘National Standards For the Industry of TCMNDT’, systematize and promote the promotion of TCMNDT.

External therapy of traditional Chinese medicine refers to using non-oral medicine to stimulate meridians, acupuncture points, skin, mucous membranes, muscles, tendons, and bones to prevent and cure diseases [3]. Modern medical research has shown that the external therapy of traditional Chinese medicine improves blood circulation, promotes the absorption and mechanization of hematoma, regulates the endocrine system, etc. Drugs penetrate the subcutaneous tissue through the skin in the affected area, and produce the relative advantage of drug concentration in the local area, reduce the local inflammatory response, promote local tissue fluid circulation, and achieve the purpose of improving symptoms [4]. The most common external therapies of traditional Chinese medicine include herbal fumigation, acupoint application, acupuncture, massage, etc [5]. Herbal acupoint application is one external therapy with Chinese characteristics, in which herbal paste is applied externally to acupoints. As a result of its practical convenience and fewer side effects, the therapy is suitable for wide application in the community [6].

Paraffin therapy has a long history in China and is performed by melting and heating medical paraffin and applying it to the surface of the body [7]. ‘Compendium of Materia Medica’ has recorded that foot frostbite applies thick fried yellow paraffin. Qi Kun, a surgical expert in the Qing Dynasty, comprehensively described the operation methods and indications of paraffin therapy in ‘Surgical Achievement’ [8]. Because of its high thermal capacity, low thermal conductivity, and long cooling time, paraffin wax is a good medium for hyperthermia conduction when in close contact with the body [9]. Paraffin therapy is a real natural therapy free of trauma, pain, and side effects. This method is simple, feasible, and inexpensive, making it among the most effective and worthy of promotion rehabilitation methods [10].

Recently, traditional Chinese medicine paraffin therapy (TCMPT) has emerged, which was based on ancient paraffin therapy, combined the mechanisms of action of various types of treatment such as meridians, acupoints, drugs, and hyperthermia from a modern medical perspective, so that it has curative properties that cannot be achieved through simple drugs, paraffin therapy, or acupuncture therapy alone [11]. Other reviews on TCMPT only review its treatment of different diseases, whereas this reviews the types of TCMPT, mechanism of action, clinical treatment, indications contraindications, and adverse events reports, to provide new ideas for the development of TCMPT based on traditional paraffin therapy and to promote the better application of traditional Chinese medicine in clinical treatment.

## Mechanisms of action and reported effects of TCMPT

The mechanism of action in external therapy of traditional Chinese medicine is mainly twofold. On the one hand, external therapy of traditional Chinese medicine promotes local blood circulation, improves immune function, and prevents and treats diseases by stimulating body surface skin (including acupoints); On the other hand, this therapy, through the transdermal drug delivery system, avoids the first-pass effect of the liver, prevents drug inactivation caused by digestive enzymes and hepatic drug enzymes, increases the body's blood concentration and evades toxic side effects of drugs on the liver and gastrointestinal tract. Therefore, the acupuncture point application method both stimulates the acupuncture point and plays an obvious pharmacological effect which has a dual therapeutic effect [12]. Xie et al. suggested that drugs acting on acupoints produce specific thermal changes making some components of drugs easier to penetrate the skin and reach deep acupoints [13]. Zhang et al. believe that herbal acupoint application in modern pharmaceutics called percutaneous drug delivery system avoids oral administration may occur liver first-pass effect and gastrointestinal inactivation improve the effective blood concentration [14].

**Fig. 1:**
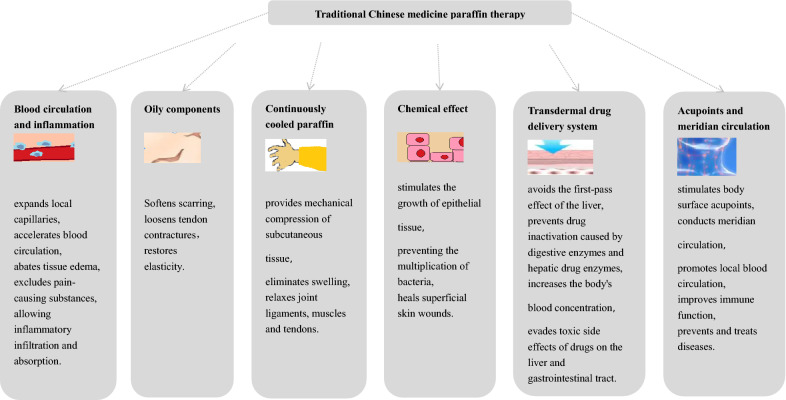
Mechanisms of action of paraffin therapy

One of the mechanisms of action of paraffin therapy is that it significantly increases microcirculation expands local capillaries and accelerates blood circulation abates tissue edema and excludes pain-causing substances allowing inflammatory infiltration and absorption to achieve the purpose of detumescence and pain relief. At the same time wax has oily components scar tendon contracture which can promote its softening and release and restore elasticity. In addition, paraffin gradually reduces its volume during cooling, and shows mechanical compression, which can prevent tissue lymph and blood exudation and enhance the absorption of exudation [10]. Wang et al. theorized that mineral oil contained in paraffin possessed a certain chemical effect on the body such as stimulating the growth of epithelial tissue and preventing bacterial reproduction which contributed to the healing of superficial skin wounds11 (Fig. 1).

## Classification of TCMPT types

Based on the relevant literature and clinical practice, this article suggests that TCMPT may be divided into five categories:

### Paraffin therapy combined with external application of herbal paste

Paraffin therapy combined with external application of herbal paste involves grinding the herb into powder, forming it into pellets with freshly squeezed ginger, maltose, or vaseline ointment, applying them to selected body parts, after heating the medical paraffin to 45–50 ℃, pouring it into a plastic bag, and then placing it on the treatment site [15, 17, 18, 23, 24, 29–31, 33, 37–39]. If the selected body parts are acupoints, it can also be called herbal acupoint application combined with paraffin therapy [20, 28].

### Paraffin therapy combined with Chinese herbal iontophoresis

Applying paraffin therapy combined with Chinese herbal iontophoresis involves the following steps: Applying paraffin therapy combined with Chinese herbal iontophoresis involves the following steps: pouring the concentrated decoction of the Chinese medicine solution onto the introduction pad using an introduction instrument and placing it on the treatment area at a temperature of 40–45 ℃. After the Chinese herbal iontophoresis, heat the paraffin block to 45–50 ℃ and then place it on this treatment site. The treatment sites of some studies are acupoints [16, 25].

### Chinese herbal paraffin block therapy

Putting the medical paraffin with a melting point of 50–55 ℃ into the paraffin box and adding the powdered Chinese herbal to dissolve it completely, then spread the dissolved Chinese herbal paraffin on a tarpaulin to make Chinese herbal paraffin block with a thickness of 2.0–3.0 cm, and then applying to the treatment site [19]. Some studies will use acupuncture, Chinese herbal fumigation, or other treatment after Chinese herbal paraffin block therapy [22, 27, 32].

### Paraffin therapy combined with Chinese herbal package

Soaking a gauze package in Chinese herbal decoction heated to 45–55 ℃ for 10–20 min. Then place the Chinese herbal package on the treatment site, put a paraffin wax cake on it and wrap it with a cotton pad [21, 26, 35, 36].

### Paraffin therapy combined with Chinese herbal collapse therapy

Putting the Chinese herbal into a non-woven bag, soaking it in warm water for 30 min, placing it in an electric constant temperature drying water tank, adjusting it to 80 ℃ for heating, and then applying it to the treatment site after the temperature has dropped to (40 ± 2) °C. After heating the medical paraffin to 45–50 ℃, place it on the treatment site [34].

## Indications

TCMPT has been used for years to prevent and treat diseases. It is beneficial for many diseases, among which internal medicine diseases include digestive system diseases (chronic gastritis [15, 16], epigastric pain [17, 18]), nervous system diseases (high levels of muscle tension of limbs in children with spastic cerebral palsy [19], diabetes peripheral neuropathy [20]). In the treatment of chronic gastritis in digestive system diseases, Chen et al. [15], based on routine nursing methods, applied the self-made TCM ‘Kunning ointment’ to the stomach and epigastric region once a day for more than 6 h. At the same time, the heated medical paraffin was placed in a 15 cm×20 cm sealing bag at a temperature of 50 ℃ and coated on the ‘Kunning Ointment’ and retained for 30 min. The results showed that herbal acupoint application combined with paraffin therapy in the treatment of chronic gastritis was significant. Wang et al. [16]used electric kerotherapy combined with Chinese herbal iontophoresis based on the control group. The electric kerotherapy acupoints were Zhongwan (CV12), Shenque (CV8), and Guanyuan (CV4), 30 min each time. The acupoints selected for Chinese herbal iontophoresis were Weishu (BL21) and Dachangshu (BL25). Methods: The concentrated decoction was evenly poured on the import pad by the imported instrument, and the temperature was 40–45 ℃. The results showed that electric kerotherapy combined with Chinese herbal iontophoresis in the treatment of chronic gastritis was remarkable, which could improve the clinical symptoms of gastric distension, gastric pain, abdominal distension, loss of appetite, and belching (Table 1).

**Table 1:** Characteristics of the Included studies

Disease name	Author	Study type	Total sample size included in article	Number of experimental group	Intervention of experimental group	Comparator	Traditional Chinese medicine used	Duration of treatment and frequency	Effective rate (Experimental group vs. Control group)	Outcome measure result
Chronic gastritis	Chen [15]	RCT	60	30	Paraffin therapy combined with external application of herbal paste	Routine care (including general care, observation of illness, diet, emotional care)	Treatment site: the stomach and epigastric region Traditional Chinese medicine: Flos Caryophylli, Rhizoma Cyperi, Radix Angelicae Dahuricae, Semen Raphani, Rhizoma Corydalis, Cattail Pollen, Fructus Schisandrae Chinensis, Radix Puerariae Lobatae, Ramulus Cinnamomi	Duration of treatment: Chinese herbal paste retained more than 6 h, paraffin retained 30 min Treatment frequency: 1 time/day, 7 days a course of treatment	96.7% vs. 86.7% (P<0.05)	Outcome measure: total effective rate Keritherapy combined with external application of Chinese medicine in the adjuvant treatment of chronic gastritis has significant clinical nursing effect
	Wang [16]	RCT	60	30	Paraffin therapy combined with Chinese herbal iontophroesis	Gastric mu- cosa protectant and gastric dynamic agent were prescribed for routine oral administration(mosapride citrate and thiosemicarbazone)	Treatment site: acupoints of paraffin therapy: Zhongwan (CV12), Shenque (CV8), Guanyuan (CV4); acupoints of Chinese herbal iontophroesis: Weishu (BL21), Changshu (BL25) Traditional Chinese medicine: Radix Astragali, Radix Angelicae Sinensis, Rhizoma Chuanxiong, Semen Persicae, Flos Carthami, Borneolum Syntheticum	Duration of treatment: 30 min Treatment frequency: 1 time/day, 15 days a course of treatment	93.3% vs. 86.7% (P<0.05)	Outcome measure: clinical symptom score In the treatment group, all of the clinical symptoms were apparently relieved or disappeared after treatment, indicating the significant difference as compared with those before treatment (P<0.05). In the control group, a part of clinical symptoms were apparently relieved. The significant difference presented in the improvement on eipgastric pain and distention as compared with those before treatment (P<0.05)
Epigastric pain	Huang [17]	RCT	64	32	Paraffin therapy combined with external application of herbal paste	Conventional therapy (acid suppression and gastric protection therapy, H. pylori eradication in H. pylori positive cases)	Treatment site: the stomach and epigastric region Traditional Chinese medicine: Pericarpium Zanthoxyli, Radix Astragali, Rhizoma Alpiniae Officinarum, Ramulus Cinnamomi, Radix Paeoniae Alba, etc	Duration of treatment: 30 min Treatment frequency: 1 time/day, 14 days a course of treatment	90.6% vs. 81.3% (P<0.05)	Outcome measure: Clinical symptom score After treatment, the scores of stomach pain, gastric distension and acid vomiting in both groups were lower than those before treatment (P<0.05, P<0.01), and the scores of each symptom in the wax therapy group were significantly lower than those in the control group (P<0.05, P<0.01)
	You [18]	RCT	60	30	Paraffin therapy combined with external application of herbal paste	Conventional treatment (acid suppressio, gastric protection, protection of gastric mucosa, etc.)	Treatment site: the stomach and epigastric region Traditional Chinese medicine: Radix Astragali, Pericarpium Zanthoxyli, Fructus Foeniculi, Ramulus Cinnamomi, Rhizoma Zingiberis	Not mentioned	96.67% vs. 76.67% (P<0.05)	Outcome measure: The TCM syndrome integral The TCM syndrome integral was decreased in both 2 groups after intervention, and was lower in the observation group than that in the control group (P<0.05)
High muscular tension of limb in children with spastic cerebral palsy	Zhang [19]	RCT	96	48	Chinese herbal paraffin cake therapy	Rehabilitation training and the treatment of Bobath	Treatment site: muscle spasm sites Traditional Chinese medicine: Radix Angelicae Sinensis, Radix Paeoniae Alba, Herba Taxilli, Pheretima	Duration of treatment: 40 min Treatment frequency: 1 time/day, 6 times/week with 1 d break. 6 weeks continuous treatment	Not mentioned	Outcome measure: Gross Motor Function Measure(GMFM), Fine Motor Function, Measure(FMFM), Modified Ashworth Scale (MAS), Clinic Spastcity Index (CSI) After treatment, scores of three functional areas of A, B, C of GMFM scale and the total score in observation group were higher than which in control group (P < 0. 05). And the scores of functional areas of B, C, D, E of FMFM scale and the total score were also higher than those in control group (P < 0. 05). Otherwise, classifications of MAS and CSI in observation group were less than those in control group (Z = − 2.337, P < 0.05 and Z = − 3.021, P < 0. 05)
Diabetes peripheral neuropathy	Wang [20]	RCT	180	90	Paraffin therapy combined with external application of herbal paste	Traditional treatment (such as Chinese herbal tonics, oral hypoglycemic drugs or insulin treatment, diet control, exercise, intravenous lipoic acid 300–600 mg, etc.)	Treatment site: Yongquan (KI1) Traditional Chinese medicine: Cortex Cinnamomi	Duration of treatment: 60 min Treatment frequency: 1–2 times / day	92.2% vs.78.8% (P<0.05)	Outcome measure: total effective rate paraffin therapy combined with herbal acupoint application of cassia paste
Supraspinatus tendinitis	Wang [21]	RCT	53	26	Paraffin therapy combined with Chinese herbal package	Lidocaine hydrochloride	Treatment site: shoulder joint Traditional Chinese medicine: Ramulus Mori, Olibanum, Myrrha, Ramulus Cinnamomi, Natrii Sulfas, Radix Achyranthis Bidentatae, Flos Carthami, Herba Lycopodii, Herba Vaccinii Urophylli, Cortex Erythrinae, Rhizoma Corydalis, Radix Angelicae Dahuricae, Radix Saposhnikoviae, Radix et Rhizoma Clematidis	Duration of treatment: 20 min Treatment frequency: 1 time/day, 4 weeks a course of treatment	NRS:92.3% vs.74.1% (P<0.05) CS:84.6% vs.70.4% (P<0.05)	Outcome measure: Numerical Rating Scalle (NRS), Activities of Daily Living (ADL), Constant-Murley Score (CS) The score of ADL were reduced, and the effect of the treatment group was better than that of the control group (P < 0.05)
Scapulohumeral periarthritis	Li [22]	RCT	60	30	Chinese herbal paraffin cake therapy and acupuncture	Shoulder joint functional exercise and oral diclofenac sodium enteric capsules	Treatment site: pain site Traditional Chinese medicine: Ramulus Cinnamomi, Ramulus Mori, Rhizoma Curcumae Longae, Radix Aconiti, Radix Aconiti Kusnezoffii, Semen Sinapis, Radix et Rhizoma Asari	Duration of treatment: 20 min Treatment frequency: 1 time/day, 10 days a course of treatment	90% vs. 80% (P<0.05)	Outcome measure: Visual Analogue Scale (VAS), Hospita for Special Surgery (HSS), Melle Score The score of HSS, score of Melle, and VAS score were all reduced, and the effect of the treatment group was better than that of the control group (P < 0.05)
	Liu [23]	RCT	96	45	Paraffin therapy combined with external application of herbal paste and massage	Acupuncture combined with massage	Treatment site: pain site Traditional Chinese medicine: Radix Aconiti, Radix Aconiti Kusnezoffii, Semen Sinapis, Olibanum, Myrrha, Radix Angelicae Pubescentis, Ramulus Cinnamomi, Radix Angelicae Dahuricae, Rhizoma Arisaematis, Agkistrodon	Duration of treatment: 40 min Treatment frequency: 1 time/day, 10 days a course of treatment, two courses of treatment in total	97.7% vs.84.3% (P<0.0025)	Outcome measure: Effective rate The effective rate in the treatment group was better than that in the control group (P < 0.025)
	Zhu [24]	RCT	560	280	Paraffin therapy combined with external application of herbal paste	Acupuncture combined with massage	Treatment site: pain site Traditional Chinese medicine: Olibanum, Radix Angelicae Pubescentis, Radix Angelicae Dahuricae, Ramulus Cinnamomi, Radix Paeoniae Rubra, Radix Aconiti, Fructus Psoraleae, Herba Vaccinii Urophylli, Herba Lycopodii, Agkistrodon	Duration of treatment: 40 min Treatment frequency: 1 time/day	99.29% vs. 92.84% (P<0.05)	Outcome measure: Effective rate The therapy of kerotherapy for relieving pain on frozen shoulder shows a good effect, and is worthy of promotion
Knee osteoarthritis	Han [25]	RCT	99	33	Paraffin therapy combined with Chinese herbal iontophroesis	Paraffin therapy or Chinese herbal iontophroesis	Treatment site: knee joint Traditional Chinese medicine: Radix Stephaniae Tetrandrae, Radix Saposhnikoviae, Radix Angelicae Sinensis, Cortex Erythrinae, Rhizoma Dioscoreae Nipponicae, Rhizoma et Radix Notopterygii, Radix et Rhizoma Clematidis, Rhizoma Chuanxiong, Olibanum, Poria, Radix Gentianae Macrophyllae, Flos Carthami, Radix Aconiti, Myrrha, Herba Vaccinii Urophylli	Duration of treatment: Chinese herbal iontophroesis retained 20 min, paraffin retained 30 min Treatment frequency: 1 time/day, 14 days a course of treatment	96.67% vs.83.33% vs.76.67% (P<0.05)	Outcome measure: The Western Ontario and McMaster Universities Osteoarthritis Index (WOMAC), McGill pain Questionnaire(MPQ) After the intervention, there was a difference between the WOMAC arthritis index and the McGill pain scale score in the test group 3 and the test group 1 and the test group 2 (P < 0.05)
	Mu [26]	RCT	64	32	Bushen Huoxue decoction and paraffin therapy combined with Chinese herbal package	Celecoxib Capsules	Treatment site: knee joint Traditional Chinese medicine: Cortex Erythrinae, Herba Vaccinii Urophylli, Olibanum, Myrrha, Radix Angelicae Sinensis, Pericarpium Zanthoxyli, Rhizoma Chuanxiong, Flos Carthami, Radix et Rhizoma Clematidis, Radix Angelicae Dahuricae, Radix et Rhizoma Glycyrrhizae,Radix Saposhnikoviae	Duration of treatment: 30 min Treatment frequency: 1 time/day, 15 days a course of treatment, 2 courses of treatment in total	97.5% vs. 78.1% (P<0.05)	Outcome measure: WOMAC The values of WOMAC after treatment of 15 d and 30 d were significantly lower than those before treatment, and the experimental group’s WOMAC was more decreased than that of the control group
	Fan [27]	RCT	78	45	Chinese herbal paraffin cake therapy and Chinese herbal fumigation	Oral administration of glucosamine potassium sulfate capsules and intra- articular injection of sodium hyaluronate	Treatment site: knee joint Traditional Chinese medicine: Radix et Rhizoma Clematidis, Semen Strychni, Rhizoma et Radix Notopterygii, Radix Arnebiae, Flos Carthami, Rhizoma Chuanxiong, Radix Angelicae Dahuricae, Radix Aconiti Brachypodi	Duration of treatment: 30 min Treatment frequency: 1 time/day, 4 weeks a course of treatment	95.56% vs. 75.76% (P<0.05)	Outcome measure: clinical symptom score, hospital for special, effective rate surgery (HSS), NRS, Matrix metallo proteinase-3 (MMP-3) assay The clinical symptom scores, NRS scores, and levels of MMP-3 were decreased, and scores of HSS were increased after treatment in the 2 groups. The effects of the treatment group was better than those of the control group (P < 0.05)
	Li [28]	RCT	60	32	Paraffin therapy combined with external application of herbal paste with “midnight noon ebb flow” theory	Paraffin therapy combined with external application of herbal paste	Comparator: Mingmen (GV4, 17:00–19:00), Shenshu (BL23,17:00–19:00); “midnight noon ebb flow” theory: Yingggu (KI10,17:00–19:00), Taixi (KI3,17:00–19:00), Fuliu (KI7,19:00–21:00) Traditional Chinese medicine: Cortex Cinnamomi, Radix Aconiti Lateralis Praeparata, Herba Epimedii	Duration of treatment: 30 min Treatment frequency: 1 time/day, 7 days a course of treatment, four courses of treatment in total	83.87% vs.72.73% (P<0.05)	Outcome measure: VAS, WOMAC After 4 weeks of intervention, the VAS scores and WOMAC scores of the two groups were lower than those before treatment (P < 0.05). The effects of the treatment group were better than those of the control group (P < 0.05)
Rheumatoid arthritis	Li [29]	RCT	80	40	Paraffin therapy combined with external application of herbal paste	Routine drug treatment (methotrexate or leflunomide) and nursing	Treatment site: painful joints Traditional Chinese medicine: Rhizoma Chuanxiong, Flos Carthami, Cortex Acanthopanacis, Rhizoma Sparganii, Borneolum Syntheticum, Lignum Sappan, Rhizoma Curcumae, Radix Aconiti Kusnezoffii, Radix Aconiti, Ramulus Cinnamomi	Duration of treatment:20- 30 min Treatment frequency: 1 time/day, 2 weeks a course of treatment	92.5% vs.85.0% (P<0.05)	Outcome measure: VAS, tenderness index, swelling index, functional classification, time of morning stiffness After treatment, the VAS score, tenderness index, swelling index, functional classification, time of morning stiffness in the 2 groups were improved than those before treatment (P < 0.05); and the treatment group was better than the control group (P < 0.05 or P < 0.01)
	Wang [30]	RCT	100	50	Traditional Chinese and Western medicine treatment (methotrexate + duhuo jisheng decoction) and paraffin therapy combined with external application of herbal paste	Traditional Chinese and Western medicine treatment (methotrexate + duhuo jisheng decoction)	Treatment site: pain sites Composition of duhuo jisheng decoction: Radix Angelicae Pubescentis, Herba Taxilli, Radix Gentianae Macrophyllae, Radix Saposhnikoviae, adix et Rhizoma Asari, Rhizoma Chuanxiong, Radix Angelicae Sinensis, Radix Rehmanniae,Radix Paeoniae Alba, Cortex Cinnamomi, Poria, Cortex Eucommiae, Radix Achyranthis Bidentatae, Radix et Rhizoma Ginseng, Radix et Rhizoma Glycyrrhizae	Not mentioned	80% vs.52% (P<0.05)	Outcome measure: MMP-3, RANKL, the baseline serum biochemical indexes After treatment, the baseline serum biochemical indexes, the levels of serum MMP-3 and RANKL both groups were significantly lower than those before treatment (P < 0.05), and the content of OPG in serum was significant- ly higher than that before treatment (P < 0.05). The changes in the observation group were more obvious than those in the control group (P < 0.05)
	Jia [31]	RCT	213	71	Paraffin therapy combined with external application of herbal paste	Conventional medications and Chinese medicine pasting therapy or conventional medications and wax therapy	Treatment site: pain sites Traditional Chinese medicine: Rhizoma et Radix Notopterygii, Radix Angelicae Pubescentis, Radix et Rhizoma Clematidis, Radix et Rhizoma Asari, Radix Aconiti, Radix Aconiti Kusnezoffii, Rhizoma Chuanxiong, Olibanum, Myrrha, Ramulus Cinnamomi, Radix Saposhnikoviae, Radix Stephaniae Tetrandrae, Radix Paeoniae Rubra, Pericarpium Zanthoxyli, Lignum Pini Nodi, Rhizoma Kaempferiae, Fructus Psoraleae, Herba Epimedii, Herba Vaccinii Urophylli, Herba Lycopodii	Duration of treatment: Chinese herbal paste retained 6–8 h, paraffin retained 20 min Treatment frequency: 1 time/week, 1 week a course of treatment	90.14% vs.73.24% vs.83.10% (P<0.05)	Outcome measure: NRS After treatment, the NRS score and morning stiffness time of the three groups were improved (P < 0.05), and the treatment group was superior to control group 1 and the control group 2 (P < 0.05)
Ankylosing spondylitis	Yu [32]	RCT	118	60	Conventional drug treatment and Chinese herbal paraffin cake therapy	Conventional drug treatment (sulfasalazine colon-soluble capsules or acetate-clofenac tablets)	Treatment site: painful joints Traditional Chinese medicine: Cortex Cinnamomi, Rhizoma Zingiberis, Semen Coicis, Sanguis Draconis, Radix et Rhizoma Asari, Radix Aconiti, Radix Aconiti Kusnezoffii, Radix et Rhizoma Salviae Miltiorrhizae, Arisaema cum Bile, Radix Dipsaci, Rhizoma Cibotii	Duration of treatment: 20–30 min Treatment frequency: 1 time/day, 6 months a course of treatment	91.67% vs 74.14% (P<0.05)	Outcome measure: bath Ankylosing Spondylitis Patient Global Score (BAS—G), Bath Ankylosing Spondylitis Disease Activity Index (BASDAI), Bath Ankylosing Spondylitis Functional Index(BASFI), VAS The BAS—G, BASDAI, BASFI, VAS scores of nocturnal pain, rachialgia and general evaluation of doctor, ESR, CPR, the time of morning stiffness, the occiput—wall distance, the finger—floor distance, the mandible—stermum distance after treatment were obviously decreased in two groups as compared with those in before treatment, and the chest expansion and Schober's test were expanded, with statistical differences (P < 0. 05)
Cervical spondylopathy	Huang [33]	RCT	64	23	Western medicine and paraffin therapy combined with external application of herbal paste	Western medicine (vincristine Injection)	Treatment site: shoulder/neck Traditional Chinese medicine: Rhizoma Chuanxiong, Semen Persicae, Flos Carthami, Radix Angelicae Sinensis, Olibanum, Myrrha, Herba Vaccinii Urophylli, Radix et Rhizoma Clematidis	Duration of treatment: 30 min Treatment frequency: 1 time/day, 7 days a course of treatment, two courses of treatment in total	93.7% vs 81.2% (P<0.05)	Outcome measure: total effective rate The average blood flow velocity of vertebral artery (VA) and basilar artery (BA) after treatment in two groups were better than those before treatment (P < 0.05 or P < 0.01), and the treatment group had more improvement than that of control group(P < 0.05)
	Qiao [34]	RCT	128	64	Paraffin therapy combined with Chinese herbal collapse therapy and massage, acupuncture, intravenous infusion of anti-inflammatory and analgesic drugs, traction conventional therapy	Chinese medicine collapse and massage, acupuncture, intravenous infusion of anti-inflammatory and analgesic drugs, traction conventional therapy	Treatment site: shoulder/neck Traditional Chinese medicine: Radix Angelicae Sinensis, Rhizoma Cibotii, Fructus Chaenomelis, Herba Lycopodii, Rhizoma Drynariae, Radix et Rhizoma Salviae Miltiorrhizae, Ramulus Cinnamomi, Herba Visci, Herba Vaccinii Urophylli, Cortex Periplocae, Flos Carthami, Rhizoma et Radix Notopterygii, Radix Angelicae Pubescentis, Radix et Rhizoma Clematidis, Herba Aristolochiae Mollissimae, Radix Aconiti, Radix Aconiti Kusnezoffii, Radix Gentianae Macrophyllae, Radix Saposhnikoviae, Rhizoma Homalomenae	Duration of treatment: Chinese herbal collapse therapy retained 20 min, paraffin retained 30 min	96.9% vs 85.9% (P = 0.03)	Outcome measure: Oswestry Disability Index(ODI) After intervention, the scores of neck and shoulder pain, limitation of neck and shoulder movement, and limitation of upper limb movement were lower in both groups than before intervention, and the difference was statistically significant (P < 0.05)
Lumbar disc herniation	Huang [35]	RCT	100	50	Routine TCM care and treatment and paraffin therapy combined with Chinese herbal package	Routine TCM care and treatment (including dehydration and decompression, qi analgesic drugs, functional exercise, etc.)	Treatment site: pain sites Traditional Chinese medicine: Radix et Rhizoma Notoginseng, Sanguis Draconis, Cortex Eucommiae, etc	Duration of treatment: 30–60 min Treatment frequency: 1 time/day, 7 days a course of treatment	96% vs 84% (P<0.05)	Outcome measure: NRS The NRS scores of the two groups after treatment were lower than those before treatment (P < 0.01), and the experimental group was lower than the control group (P < 0.01)
	Huang [36]	RCT	158	79	Routine TCM care and treatment and paraffin therapy combined with Chinese herbal package	Routine TCM care and treatment (including medication, life coaching, rehabilitation training, psychological intervention)	Treatment site: pain sites Traditional Chinese medicine: Radix et Rhizoma Notoginseng, Sanguis Draconis, Cortex Eucommiae, etc	Duration of treatment: 30–60 min Treatment frequency: 1 time/day, 7 days a course of treatment	Not mentioned	Outcome measure: the Quality of Life, Health Survey Scores,Lumbar Disc Function After treatment and care, the quality of life and health survey scores and lumbar disc function of the two groups were compared, and the experimental group was significantly increased, with statistically significant difference (P < 0.01). After treatment, the skin temperature and inflammatory reaction in the experimental group were significantly reduced, and the difference was statistically significant (P < 0.01)
Thoracolumbar compression fracture	Liu [37]	RCT	170	90	Routine treatment and paraffin therapy combined with external application of herbal paste	Routine treatment (including life coaching, rehabilitation training, TDP lamp irradiation)	Treatment site: pain sites Traditional Chinese medicine: Cortex Phellodendri Chinensis, Radix Scutellariae, Semen Persicae, Flos Carthami, Olibanum, Myrrha, etc	Duration of treatment: Chinese herbal paste retained overnight, paraffin retained 20–30 min Treatment frequency: 1–2 times/day(paraffin therapy), 4 weeks of treatment in total	92.2% vs 80.0% (P<0.05)	Outcome measure: total effective rate The total effective rate of the observation group was significantly higher than that of the control group, and the difference was statistically significant (P < 0.05)
Distal radius fracture	Li [38]	RCT	80	40	Plaster external fixation with early opening combined with paraffin therapy combined with external application of herbal paste	Plaster external fixation, combined with PTCWEAOHP after removal of plaster	Treatment site: fracture site Traditional Chinese medicine: Rhizoma Drynariae, Radix Dipsaci, Rhizoma Corydalis, Rhizoma Sparganii, Herba Lycopodii, Radix Angelicae Sinensis	Duration of treatment: 30 min Treatment frequency: 1 time/day, 4 weeks of treatment in total	Not mentioned	Outcome measure: VAS, Cooney score The difference in VAS scores between the 2 groups was statistically significant (*P* < 0.05) when functional exercise was performed immediately after removal of the cast, and the pain level in the treatment group was less than that in the control group. The difference between the two groups was statistically significant (P < 0.05), and the treatment group had better wrist function Cooney Score than the control group
Patellar fracture	Song [39]	RCT	150	50	Paraffin therapy combined with external application of herbal paste combined with shape memory alloy patella claw treatment	Chinese medicine hot washing combined with shape memory alloy patella claw treatment or Chinese medicine hot washing combined with patellar wire internal fixation treatment	Treatment site: pain sites Traditional Chinese medicine: Sanguis Draconis, Olibanum, Myrrha, Radix et Rhizoma Notoginseng, Eupolyphaga seu Steleophaga, Aspongopus, Radix Dipsaci, Cortex Acanthopanacis, Rhizoma Zingiberis Recens, Herba Artemisiae Anomalae, Flos Carthami, Pyritum, Borax, Semen Strychni, Borneolum Syntheticum, cucumber seed, Whole chicken bone	Duration of treatment: 30 min Treatment frequency: 1 time/day, 10 days a course of treatment, 2 courses of treatment in total	92.87% vs 89.71% vs 87.50% (P<0.05)	Outcome measure: knee mobility The Knee Mobility of experimental group was better than control group 1 and control group 2, and the difference was statistically significant (P < 0.05)

*RCT* Randomized Controlled Trial

In the treatment of epigastric pain in digestive system diseases, Huang et al. [17] on the basis of the control group treatment, applied a block of self-made ‘warming stomach prescription’ to the stomach and epigastric region. Then the medical paraffin was heated to 45–50 ℃, poured into the plastic bag, and placed on the TCM block for external application, 30 min each time. The results showed that herbal acupoint application combined with paraffin therapy is a simple, effective, safe, simple and easy-to-use treatment method with no obvious adverse effects, and is worthy of clinical promotion. You et al. [18] on the basis of the control group treatment, applied the TCM ‘pain-relieving ointment’ to the stomach and epigastric region. Then the medical paraffin was heated to 45–50 ℃, poured into the plastic bag, coated on the ‘pain-relieving ointment’ and covered with a small blanket to keep the area warm. The results showed that herbal acupoint application combined with paraffin therapy can effectively relieve the discomfort symptoms of patients with gastric pain.

Surgical diseases include chronic soft tissue injury disease (supraspinatus tendinitis [21], scapulohumeral periarthritis [22–24]), bone and joint diseases(knee osteoarthritis [25–28], rheumatoid arthritis [29–31], ankylosing spondylitis [32], cervical spondylopathy [33, 34], lumbar disc herniation [35, 36]), and orthopedic diseases(thoracolumbar compression fracture [37], distal radius fracture [38], patellar fracture [39]).

## Age of patients using TCMPT

According to the included literature, except for the literature on children’s diseases, most of the patients included in the literature are 18–75 years old, and some special diseases (such as thoracolumbar compression fracture) will increase the age to more than 80 years old; among them, patients with rheumatoid arthritis, knee osteoarthritis and lumbar disc herniation who received TCMPT were mostly over 40 years old. It can be seen that TCMPT is suitable for people of all ages, but for people under 18 years old and over 75 years old, it is necessary to pay attention to the types of diseases used in TCMPT (Table 2).

**Table 2:** Age of Patients Using TCMPT

Disease name	Author	Age	Inclusion criteria	Exclusion criteria
Chronic gastritis	Chen [15]	36–60 years		Under 18 years and over 70 years
	Wang [16]	22–60 years		Under 18 years and over 75 years
Epigastric pain	Huang [17]	24–62 years	20–60 years	
	You [18]	46–77 years		
High muscular tension of limb in children with spastic cerebral palsy	Zhang [19]	12–36 months	12–36 months	
Diabetes peripheral neuropathy	Wang [20]	Median age: 69 years		
Supraspinatus tendinitis	Wang [21]	26–70 years		
^Scapulohumeral periarthritis^	Li [22]	40–65 years		
	Liu [23]	45–70 years		
	Zhu [24]	42–71 years		
Knee osteoarthritis	Han [25]	40–75 years		
	Mu [26]	48–65 years		
	Fan [27]	42–75 years		
	Li [28]	40–75 years		
Rheumatoid arthritis	Li [29]	21–69 years	20–70 years	
	Wang [30]	54–52 years	35–70 years	
	Jia [31]	34–65 years	18–65 years	
Ankylosing spondylitis	Yu [32]	21–47 years	18–50 years	
Cervical spondylopathy	Huang [33]	None		
	Qiao [34]		29–70 years	
Lumbar discherniation	Huang [35]	40–78 years		
	Huang [36]	34–67 years		
Thoracolumbar compression fracture	Liu [37]	24–84 years		
Distal radius fracture	Li [38]	51–75 years		
Patellar fracture	Song [39]		20–60 years	

## Contraindications

TCMPT is contraindicated directly on skin inflammation, any skin lesion, eyes, lymph nodes, or varicose veins. Patients with cancer, as well as those with serious diseases of the heart, liver, brain, kidney, etc., are contraindicated [40, 41]. It is also contraindicated in patients who have pacemakers or suffer from hemophilia. An acute infection, the use of anticoagulants, bleeding disorders, severe heart conditions and pacemakers, pregnancy, puerperium, menstruation, anemia, medical problems, allergic reactions to topical medications, and hypersensitive skin are all contraindications to TCMPT [15, 42].

## Adverse events

TCMPT is a form of combination therapy. The following adverse events (AEs) have been reported with this therapy:

In general, external therapy of traditional Chinese medicine is relatively safe and AEs are relatively rare. Majority of AEs are mild or moderate in severity [43]. The most common AEs to herbal acupoint application are skin redness, itchiness, tingling, congestion, rash, etc [44–46].An important factor is that the patient's skin is allergic to the herb or tape. Blisters and ulcers may form if the treatment is applied too long. Li et al. [47] reported a case of paraffining burns, in which the patient's right knee was burned owing to the lack of awareness of the health provider. Wang et al. [47] found that treatment groups that used paraffin therapy had arisen skin diseases (skin allergies), but did not have vomiting, scalds, respiratory failures, heart failures, or deaths. This article summarized the treatment-related adverse events that occurred during the trial [47] (Table 3).

**Table 3:** Classification of adverse events

Adverse events			
*n* (%)	Exp (n = 27)	Con (n = 25)	Total (n = 52)
Increased blood pressure	2 (7.4)	5 (20)	7 (13)
Vomiting	0 (0)	0 (0)	0 (0)
Increased pain	0 (0)	15 (60)^a^	15 (29)
Scald	0 (0)	0 (0)	0 (0)
Skin disease	1 (3.7)	0 (0)	1 (2)
Deaths	0 (0)	0 (0)	0 (0)

^a^*P* < 0.05

## Infection control measures

Many articles mentioned that the most common adverse reaction caused by paraffin therapy burns. To reduce the occurrence of burns, paraffin should be cooled to the appropriate temperature before treating the patient, the patient should be asked how he/she feels at any time during the treatment process and the patient’s skin should be observed, if erythema, blisters, scratching, etc. should be stopped immediately; cold water should be avoided after the treatment [20]. If the burn wound has purulent secretions, the wound needs to be cleaned and the infection controlled by thoroughly flushing the wound with 3% hydrogen peroxide solution, then rinsing the wound with 0.9% saline and applying topical burn ointment locally; if the wound has blisters, small blisters with iodophor disinfection, saline rinse, topical burn ointment; large blisters washed with saline, iodop hor disinfection, with 5 ml sterile syringe to extract the blister liquid, topical burn ointment [48].

## Summary

In conclusion, TCMPT, which combines meridians and acupoints, drugs, and hyperthermia, has been very effective in some diseases. We reviewed a new classification of TCMPT, mechanism of action, clinical treatment, indications contraindications and adverse events reports to provide new ideas for the development of TCMPT based on traditional paraffin therapy and to promote the better application of traditional Chinese medicine in clinical treatment. This article suggested that TCMPT can promotes local blood circulation, improves immune function, relaxes joint ligaments, muscles and tendons, evades toxic side effects of drugs on the liver and gastrointestinal tract, excludes pain-causing substances allowing inflammatory infiltration and absorption, and prevent tissue lymph and blood exudation but also to enhance the absorption of exudate, and loosens tendon contractures, restores elasticity; it is divided into five categories, namely paraffin therapy combined with external application of herbal paste, paraffin therapy combined with Chinese herbal iontophroesis, Chinese herbal paraffin block therapy, paraffin therapy combined with Chinese herbal package, paraffin therapy combined with Chinese herbal collapse therapy. The most common AEs to TCMPT are skin diseases (including skin redness, itchiness, tingling, congestion, rash). It can be seen from the included literatures that TCMPT can be used to treat digestive system diseases, nervous system diseases, chronic soft tissue injury disease, bone and joint diseases, and orthopedic diseases. But these literatures are all Chinese, and most of them are not of high quality. Therefore, attention should be paid to improve the quality of literature in future related trials (including clarifying blinding and adding descriptions related to adverse effects, etc.). If we want to vigorously promote TCMPT, the types of paraffin, the treatment sites for different diseases, and the size of ointments made of Chinese herbs need to be standardized. At the same time, TCMPT as part of Chinese medicine treatment, personalized treatment is also one of its characteristics, for example, the choice of type and dosage of Chinese herbs, differences in individual treatment sites, frequency of treatment, etc., all require us to develop specific treatment protocols according to the patient’s situation. Also, TCMPT is still mainly used for the treatment of surgical diseases, and it is not widely used in clinical diseases, and few people understand and apply it. Therefore, we need to promote TCMPT more recently and apply it to more kinds of diseases, so as to provide new treatment methods for different diseases.

## Data Availability

Because of the confidentiality of the individuals included in the study, the data underlying this article cannot be shared publicly. Data will be made available upon reasonable request to the corresponding author.

## References

[1] Haiping P, Huifang Z, Jie M (2011). A review of non-oral drug delivery methods for asthma treatment in Chinese medicine. China Med Herald.

[2] Healthy China Action Promotion Committee. Healthy China Action 2021–2022 Assessment implementation plan. China Government Network. 2022.02.08, (1).

[3] Qing Z, Guozhi H, Donghui L (2010). Progression of external treatment with traditional Chinese medicine on fracture. J Liaoning Univ Chin Med.

[4] Weicheng X, Hong J, Jun M (2014). Current status of oedema treatment with Chinese herbal external treatment methods. Chin J Tradit Chin Med.

[5] Qingwen Z (2010). Discussion on several key issues in the development of external therapy of traditional Chinese medicine. J Extern Ther Tradit Chin Med.

[6] Xijian L, Tao H (2014). Current status and ideas of research on Chinese medicine acupressure. Chin Med Inf.

[7] Wanlin L, Yunlan J, Tingting Z (2016). Meta-analysis of the efficacy of wax therapy for cervical spondylosis. West Med.

[8] Fenghong G, Peng F, Xun Z, Huanwei Y, Lei X (2018). New advances in the clinical application of wax therapy. Chin Gen Med.

[9] Zhihong L, Huaxin W (2017). Current status of the application of Chinese medicine waxing technology in clinical practice. Gen Pract Nurs.

[10] Qingfu B (2007). The role of wax therapy and its clinical application. J Pract Med.

[11] Ye W, Fusheng L (2015). Observation on the efficacy of Chinese medicine wax therapy combined with ultrashort wave in the treatment of knee synovitis. Chin J Tradit Chin Med Inf.

[12] Bao Z, Ruiping S, Yanjun Z (2016). Discussion on the theory and mechanism of Chinese medicine acupuncture point application therapy. Gansu Med.

[13] Yang X, Xueqing Y (2008). Experimental description of the mechanism of action of acupressure and its clinical application. Chin Med Guide.

[14] Xiaoming Z, Qiaoling P (2005). The mechanism of action of acupuncture point application therapy. Chin Folk Ther.

[15] Yan C, Zhiping F (2016). Keritherapy combined with external application of Chinese medicine in the adjuvant treatment of chronic gastritis for 30 cases. Guangming J Chin Med.

[16] Wang X, Yixiu C (2014). Efficacy observation of electric kerotherapy and chinese herbal iontophoresis in the auxiliary treatment of chronic gastritis. World J Integr Tradit West Med.

[17] Bei H (2017). Clinical observation of wax therapy of traditional Chinese medicine for epigastric pain of spleen-stomach deficiency-cold. Research of integrated. Tradit Chin West Med.

[18] Shuru Y, Ping Z (2020). Application of wax therapy combined with Traditional Chinese Medicine external application for stomachache patients with spleen-stomach deficiency-cold syndrome. Nurs Integr Tradit Chin West Med.

[19] Ruiyuan Z, Qingyun B (2019). Clinical observation on traditional Chinese medicine wax therapy combined w ith bobath in the treatment of motor function of children w ith spastic cerebral pals. Chin Med Mod Distance Edu China.

[20] Zhen W (2019). Nursing care of patients with diabetes peripheral neuropathy treated by acupoint application of cassia wax paste. Health Prot Promot.

[21] Ye W, Yichen B (2016). Chinese medicine paraffin combined with massage treat supraspinatus tendonitis. J Changchun Univ Chin Med.

[22] Qiang Z, Qiang Z, Jianzhong G, Ye W (2016). Clinical observation on treating 280 cases of frozen shoulder by the therapy of kerotherapy for reliving pain. Clin J Chin Med.

[23] Xiangyang L (2005). Clinical observation on treating 96 cases of scapulohumeral periarthritis by wax therapy of traditional Chinese medicine combined with massage. Chin Manip Rehabilit Med.

[24] Zhe L (2018). Opposing needling combined with wax therapy of traditional Chinese medicine in the treatment of 30 cases of scapulohumeral periarthritis of wind-cold-damp type. Henan Traditi Chin Med.

[25] Xiaoyu H. Effect of wax therapy combined with iontophoresis of traditional Chinese medicine on the nursing effect of patients with knee osteoarthritis [Master], Heilongjiang University of Chinese Medicine; 2018.

[26] Dinghai M, Zuyao D, Xiangsong L (2022). Clinical Study of bushen huoxue decoction combined with Chinese herbal wax therapy on the treatment of postmenopausal knee osteoarthritis of kidney deficiency and blood stasis. Chin J Tradit Med Traumatol Orthop.

[27] Huixia F, Zhili F, Wei Z (2021). Clinical study on Chinese herbal fumigation and paraffin therapy combined with routine western medicine for knee osteoarthritis. New Chin Med.

[28] Suqian L, Yu L, Huaxin W (2019). Effect of midnight noon ebb flow theory applying in wax therapy of traditional Chinese medicine on knee osteoarthritis. Guid J Tradit Chin Med Pharm.

[29] Feiyan L, Ting L (2017). Effect of Chinese-medicine Wax therapy on the clinical efficacy of rheumatoid arthritis. Rheum Arthritis.

[30] Jing W, Rui W (2018). Clinical efficacy of Chinese medicine paraffin in treatment of rheumatoid arthritis patients with wind-cold and dampness syndromes and the change and significance of serum MMP-3, OPG and RANKL. Chin Arch Tradit Chin Med.

[31] Xi-jiao J, Guo-sheng Z (2018). Clinical observation on 71 cases of rheumatoid arthritis treated with wax therapy combining Chinese medicine pasting therapy and conventional drug. Rheum Arthritis.

[32] Shujing Y (2016). Clinical observation of Chinese paraffin paste combined with early nursing care on the treatment of active ankylosing spondylitis. Hebei J Tradit Chin Med.

[33] Bei H (2017). Clinical observations of wax therapy in treatment of vertebral artery type of cervical spondylosis. Chin J Clin Ration Drug Use.

[34] Qingcui Q, Haoying D (2021). Effect observation of application of modified traditional Chinese medicine collapsing method combined with wax therapy in the treatment of cervical spondylotic radiculopathy. Chin Evid-Based Nurs.

[35] Yu H, Wenfeng Y, Zhelin L, Yuqiu Z, Emei N (2016). Effect observation of kerotherapy on remission of patients with qi stagnation and blood stasis type of lumbar disc herniation. Nurs Integr Tradit Chin West Med.

[36] Yu H, Wen-feng Y, Zhe-lin L (2017). Effect of Chinese medicine keritherapy on quality of life in patients with TCM qi stagnation and blood stasis type lumbar disc herniation. Chi Family Medi.

[37] Fanming L (2010). Effect observation and nursing of 90 cases of thoracolumbar compression fracture treated with external application of traditional Chinese medicine combined with wax therapy. Journal of Qilu Nurs.

[38] Rui L, Yunling G, Sudan Z (2017). Effect of early fenestration with plaster external fixation combined with traditional Chinese medicine wax therapy on type C fracture of distal radius in middle-aged and elderly patients. Hebei J Tradit Chin Med.

[39] Zhenjiang S, Yan Z, Yejin F (2008). Wax therapy of traditional Chinese medicine combined with shape memory alloy patellar claw in the treatment of patellar fracture. Guangming J Chin Medi.

[40] Jing G, Yan Y, Chenxi W (2017). Acupoint plaster therapy with midnight–noon ebb–flow hour–prescription method for senile osteopo–rosis: a randomized controlled trial. Chin Acupunct.

[41] Libing Z, Wei Z, Vivian W (2016). Randomized trial of acupoints herbal patching in Sanfu Days for asthma in clinical remission stage. Clin Transl Med.

[42] Yin S, Li H, Cuihong Z (2020). Efficacy of acupuncture at three nasal acupoints combined with acupoint application for perennial allergic rhinitis: a multicenter, randomized controlled trial protocol. Trials.

[43] Shanshan C (2017). Clinical observation of acupoint therapy combined with Decoction for functional dyspepsia of liver depression and qi stagnation type. Guangming J Chin Med.

[44] Hongli Z, Minghui Z, Lixin F (2016). Observation on short-term efficacy and adverse reactions of Sanfu plaster in treatment of allergic rhinitis. Chin Acupunct Moxib.

[45] Lizhen T, Wenqiang Z, Fengzhi L, Xiaolan C, Bing O (2020). Clinical observation of pricking sifeng (EX-UE10) combined with acupoint application for Infantile functional dyspepsia. Med Forum.

[46] Dehua L, Xie J, Yulan R, Xie J, Hui Z, Junling L (2021). Effectiveness and safety of acupoint application of Guan Xin Su He Pill for patients with chronic stable angina pectoris: a multi-center, randomized controlled trial. Chin J Integr Med.

[47] Jing W, Peng Y, Ming Z, Xudong G, Yan L, Mingyue X (2017). Reduction in spasticity in stroke patient with paraffin therapy. Neurol Res.

[48] Jianhua N (2015). Wound care of a diabetic patient with scalded feet co-infected with infection. J Nurs Contin Educ.

